# African spiny mice show resistance to DMBA/TPA-induced squamous carcinogenesis with distinct benign tumor profile

**DOI:** 10.1093/procel/pwaf024

**Published:** 2025-03-22

**Authors:** Fathima Athar, Francesco Morandini, Iqra Fatima, Isabella Silvestri, Sei Joong Kim, Minseon Lee, Xiaoyan Liao, Andrei Sharov, Vladimir Botchkarev, Andrei Seluanov, Vera Gorbunova

**Affiliations:** Department of Biology, University of Rochester, Rochester, NY 14627, United States; Department of Biology, University of Rochester, Rochester, NY 14627, United States; Department of Dermatology, Boston University, Boston, MA 02118, United States; Department of Biology, University of Rochester, Rochester, NY 14627, United States; Department of Biology, University of Rochester, Rochester, NY 14627, United States; Department of Biology, University of Rochester, Rochester, NY 14627, United States; Department of Pathology and Laboratory Medicine, University of Rochester Medical Center, Rochester, NY 14642, United States; Department of Dermatology, Boston University, Boston, MA 02118, United States; Department of Dermatology, Boston University, Boston, MA 02118, United States; Department of Biology, University of Rochester, Rochester, NY 14627, United States; Department of Biology, University of Rochester, Rochester, NY 14627, United States

**Dear Editor,**

African spiny mouse, *Acomys dimidiatus*, is a mammalian model for regeneration studies because of its ability to functionally regenerate several tissues. As limited regenerative abilities in mammals are viewed as an antitumor strategy, it is intriguing how *Acomys* balances regeneration and tumor suppression. In this study, we investigated if *Acomys* is susceptible to carcinogenesis. We report that, like in mice, two oncogenic hits—activation of oncogenic Ras^G12V^ and inactivation of p53 or pRb—are sufficient to malignantly transform skin fibroblasts from *Acomys*. However, *in vivo*, *Acomys* showed resistance to DMBA/TPA-induced skin carcinogenesis. Tumor formation in *Acomys* was significantly delayed relative to C57BL/6 mice. Furthermore, a longer TPA treatment period of 30 weeks *Acomys* induced benign sebaceous adenomas while C57BL/6 mice formed pre-malignant carcinoma just after 22 weeks, indicating that *Acomys* is resistant to squamous skin carcinogenesis typically induced by DMBA/TPA. *Acomys* showed stronger upregulation of immune response and higher apoptosis in DMBA/TPA-treated skin when compared with mice. Wnt/β-catenin signaling, a major mediator of squamous carcinogenesis, was inhibited in upon DMBA/TPA treatment in *Acomys*. Overall, our study shows that despite high regenerative capacity *Acomys* evolved compensatory mechanisms to suppress tumorigenesis that include enhanced immune response to oncogenic stimuli, higher apoptosis, and inhibition of Wnt/β-catenin signaling.

When it comes to tissue regeneration in mammals, African spiny mice, *Acomys*, have a celebrity status. These are the only mammals capable of remarkable non-fibrotic regeneration and repair in multiple tissues. Scarless tissue regeneration and repair in *Acomys* is documented in studies involving full excision skin wounds, ear punches, kidneys, spinal cord, heart, and skeletal muscle (Tomasso et al., 2024).

With rising cancer incidences in human population, finding preventive measures and cures for cancer remains the holy grail of biology research. So far, the comparative biology approach to study cancer has brought forth several naturally occurring and evolutionarily selected anti-cancer mechanisms to light. Multiple cell-intrinsic or cell autonomous, and cell microenvironment-mediated or cell-non-autonomous adaptations have been uncovered in long-lived species. These include the higher dosage and/or activity of tumor suppressors like p53 in elephants, downregulation of telomerase activity in large species, enhanced DNA repair in long-lived rodents, regulation of uncontrolled proliferation by early contact inhibition in naked mole rats, and transposon-mediated clearance of premalignant cells in blind mole rats (Zhao et al., 2021).

With a lifespan of around 5–6 years, *Acomys* lives twice as long as laboratory mice. Hence, *Acomys* has twice longer time to develop cancer. However, there are no records of spontaneous tumor development in either wild-caught or in animal colonies of research labs. Recent attempts to generate iPSCs from neonatal dorsal skin-derived fibroblasts of *Acomys* revealed that tumor suppressors may be involved in restriction of dedifferentiation, leading authors to speculate if *Acomys* may have unreported resistance to cancer (Sandoval et al., 2022). These observations make it intriguing to uncover how *Acomys* maintains its tissue plasticity while keeping malignant transformation at bay.

The number of oncogenic “hits” required to transform a cell type provide clues into an organism’s inherent barriers to transformation. Generally, the number of oncogenic hits required increases with body size and lifespan in rodents (Tian et al., 2018). Mouse fibroblasts require two oncogenic hits—inactivation of p53 or pRb and activation of HRAS. On the other hand, human fibroblasts require 5—inactivation of p53, pRb, and PP2A and activation of HRAS and telomerase (Rangarajan et al., 2004). Naked mole rat fibroblasts require the inactivation of both p53 and pRb in addition to the depletion of hyaluronan (Tian et al., 2013).

To investigate the number of oncogenic hits that would transform skin fibroblasts of *Acomys*, we generated transformed cells stably expressing HRAS^G12V^, Simian Vacuolating virus 40 Large T antigen (SV40 LT), and two mutants of SV40 LT, SV40 LT-K1, and SV40 LT-Δ 434–444 in various combinations. HRas^G12V^ is a constitutively active mutant of HRAS. SV40 LT, an oncoprotein can bind and inactivate p53 and pRb family of proteins. The mutant—SV40 LT-K1 inactivates p53 only, and SV40 LT-Δ 434–444 inactivates pRb family only. The expression of oncogenic HRAS^G12V^ and SV40 LT constitutes three oncogenic hits: first, overexpression of oncogenic HRAS; second, inactivation of p53; and third, inactivation of pRb. Expression of the mutants with HRAS^G12V^ constitutes two oncogenic hits. We did not overexpress TERT as our Telomere Repeated Amplification Protocol (TRAP) assay with *Acomys* fibroblasts showed presence of telomerase activity (Fig. S1).

First, we tested anchorage-independent growth of transformed cells in a soft agar assay. Overexpression of HRAS^G12V^ along with either of SV40 LT mutants was sufficient for colony formation in soft agar (Fig. 1A). This suggested that two oncogenic hits were sufficient to transform skin fibroblasts from *Acomys*, just like laboratory mice. Fibroblasts overexpressing HRAS^G12V^-only or GFP only did not form colonies in the assay.

**Figure 1.**
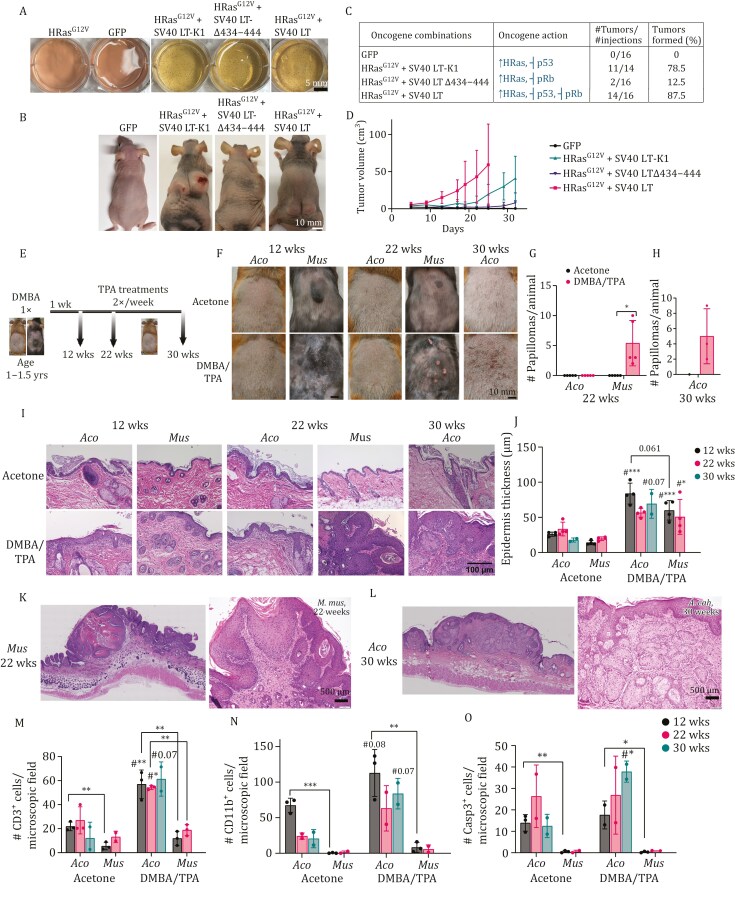
***In vitro* and *in vivo* carcinogenesis in *Acomys***. (A) Representative image of soft agar assay with transformed skin fibroblasts of *Acomys* stably expressing oncogenes in combinations indicated. Colonies in the wells of a 6-well plate stained using nitroblue tetrazolium chloride solution (NBT) are shown. Scale bar, 5 mm. (B) Xenograft assay: Representative images of nude mice injected with transformed skin fibroblasts of *Acomys* stably expressing oncogenes in combinations indicated. Scale bar, 10 mm. (C) Table showing percentages of tumor formation in nude mice injected with transformed skin fibroblasts of *Acomys* stably expressing oncogenes in the combinations indicated. (D) Tumor volumes from the xenograft assay are plotted to show tumor growth curves. Error bars indicate SD. (E) Schematic showing DMBA/TPA treatment schedule in C57BL/6 mice and *Acomys*. (F) Representative images of DMBA/TPA-treated dorsal skin in *Acomys* (*Aco*) and C57BL/6 mice (*Mus*) at 12, 22, and 30 weeks of TPA treatment. Scale bar, 10 mm. (G) Quantification showing the number of papillomas/animal formed at the end of week 22 in C57BL/6 mice and *Acomys*. Error bars show standard deviation (SD). Two-tailed unpaired *t*-tests were performed for comparisons within species (indicated by #, acetone vs. DMBA/TPA) and between species (*Aco* vs. *Mus*, DMBA/TPA-treated). (H) Quantification showing the number of papillomas/animal formed at the end of 30 weeks in *Acomys*. Error bars show standard deviation (SD). Two-tailed unpaired *t*-tests were performed for comparisons within species (indicated by #, acetone vs. DMBA/TPA) and between species (*Aco* vs. *Mus*, DMBA/TPA-treated). (I) H&E staining of DMBA/TPA-treated skin of *Acomys* and C57BL/6 mice after 12, 22, and 30 weeks of TPA treatment. Scale bar, 100 μm. (J) Quantification of epidermal thickness (μm) from H&E sections using Image J. Error bars show standard deviation (SD). Two-tailed unpaired *t*-tests were performed for comparisons within species (acetone vs. DMBA/TPA) and between species (*Aco* vs. *Mus*, DMBA/TPA-treated). (K) (Left) Representative H&E staining to show whole papilloma at 22 weeks in DMBA/TPA-treated mice skin, generated my merging individual scans of entire section. (Right) Representative H&E staining showing histology of squamous papillomas in mice at 22 weeks. Scale bar, 500 μm. (L) (Left) Representative H&E staining to show the whole papillomas at 30 weeks in DMBA/TPA-treated *Acomys* skin, generated by merging individual scans of entire section. (Right) Representative H&E staining showing histology of squamous papillomas in *Acomys* at 30 weeks. Scale bar, 500 μm. (M–O) Quantification of immunohistochemistry for CD3 (M); CD11b (N) and Caspase 3 (O) positive cells in acetone and DMBA-treated skin of *Acomys* and C57BL/6 mice. Two-tailed unpaired *t*-tests were performed for comparisons within species (indicated by #, acetone vs. DMBA/TPA) and between species (*Aco* vs. *Mus*, DMBA/TPA-treated). *P < 0.05, **P < 0.01, ***P < 0.001.

To check if these transformed cells can form tumors, we performed a xenograft assay in nude mice. We injected nude mice with fibroblasts overexpressing GFP (control) or the transformed stable cells expressing HRAS^G12V^, SV40 LT, and its mutants in the three oncogene combinations described earlier. Overall, we performed 16 injections (8 per biological replicate). Though transformed cells of all three oncogene combinations formed tumors (Fig. 1B), the time taken (Fig. 1D), and the percentage of tumors formed (Fig. 1C) varied and was in the following order—HRAS^G12V^ + SV40 LT (87% tumors) > HRAS^G12V^ + SV40 LT-K1 (78%) > HRAS^G12V^ + SV40 LT-Δ 434–444 (12.5%) (Fig. 1C and 1D). *Acomys* fibroblasts expressing GFP control did not form any tumors in nude mice. The least number of tumors were formed in HRAS^G12V^ + SV40 LT-Δ 434–444 where p53 is active. This suggests that p53 has relatively higher importance than pRb in preventing malignant transformation in *Acomys* fibroblasts.

Since cell cycle arrest and cellular senescence are important for preventing tumorigenesis, we investigated these responses in *Acomys* skin fibroblasts. Skin fibroblasts from *Acomys*, C57BL/6 mice and wild-caught *M. musculus* mice were irradiated with two doses of γ-radiation, 10 Gy and 20 Gy. *Acomys* fibroblasts showed efficient cell cycle arrest, at both 10 and 20 Gy (Fig. S2A), and higher levels of apoptosis at 20 Gy when compared with mice (Fig. S2B). These observations were corroborated by changes in the transcriptome of radiation-treated fibroblasts (Data S1). *Acomys* showed significant upregulation of biological processes like “apoptosis,” “tissue remodeling,” and “protein degradation,” which were not significantly upregulated in mice. Moreover, processes related to cell cycle progression were strongly downregulated in *Acomys*, while this was not significant in mice (Fig. S2C; Data S2). This indicates that in response to stress, *Acomys* has a tighter cell cycle control and eliminates damaged cells by triggering cell death. At day 12 post-irradiation, the number of senescence-associated β-galactosidase (SA-β-gal) positive cells was higher in *Acomys* when compared with mice at 20 Gy radiation dose (Fig. S2D and S2E). Transcript levels of *Cdkn1a* at both 24 h and 12 days were higher in radiation treated *Acomys* fibroblasts when compared with laboratory mice (Fig. S2F). We used published gene set signatures of senescence (Data S3), including SenMayo gene sets, to analyze Senescence-associated secretory phenotype (SASP) in radiation-treated fibroblasts. At 12 days post-radiation both *Acomys* and mice showed comparable SASP profile. However, sub-categorized SASP gene sets like “NFκB regulated SASP,” “fibroblast SASP factors,” “intercellular signal molecule,” and “MMP” (matrix metalloproteinases) were more significantly enriched in radiation-treated *Acomys* fibroblasts when compared with mice fibroblasts (Fig. S2G). These results indicate that the response of radiation-treated *Acomys* fibroblasts may be more robust than laboratory mice.

We next tested whether *Acomys* is susceptible to *in vivo* carcinogenesis using the two-stage chemical skin carcinogenesis model. *Acomys* and C57BL/6 mice, both aged 1–1.5 years, were treated one time with tumor initiator, DMBA (7,12-dimethylbenz[a]anthracene) dissolved in acetone and topically applied on the shaved dorsal skin. Tumor promoter, TPA (12-O-tetradecanoylphorbol-13-acetate, also dissolved in acetone) treatments, topically applied to the skin beginning 1 week after DMBA treatment, were carried out twice a week for 30 weeks. Animals were sacrificed at 12, 22, and 30 weeks (Fig. 1E). At week 12, *Acomys* skin treated with DMBA/TPA looked similar to that of acetone-treated skin. However, the skin of C57BL/6 mice treated with DMBA/TPA looked highly distressed with palpable thickening. At the end of 12 weeks, both DMBA/TPA-treated *Acomys* and C57BL/6 mice did not develop visible skin tumors (Fig. 1F). By week 22, C57BL/6 mice developed visible tumors, with an average of about 5 tumors/mouse (Fig. 1F and 1G). However, *Acomys* skin treated with DMBA/TPA neither developed tumors nor appeared distressed at this time point (Fig. 1F and 1G). We continued treating *Acomys* with TPA/acetone for an additional 8 weeks and observed that *Acomys* developed smaller but visible tumors by week 30 with a similar average of 5 tumors/animal (Fig. 1F and 1H).

We sacrificed animals at 12, 22, and 30 weeks of DMBA/TPA treatment and assessed histology of the skin (Fig. 1I). First, we checked if the skin tumors formed in both species were histologically similar. We discovered that while C57BL/6 mice formed squamous papilloma with marked cytologic atypia consistent with squamous cell carcinoma *in situ* at week 22, *Acomys* formed sebaceous adenomas, a benign sebaceous gland tumor, at week 30. Of the five lesions present in the skin sections of C57BL/6 mice, two showed squamous dysplasia/carcinoma *in situ*, while the other three were papillomas with cytologic atypia—both precursors to squamous cell carcinoma (Fig. 1K). In *Acomys*, 5/6 tumors were benign sebaceous adenomas (Fig. 1L). Epidermis adjacent to the tumors in both species showed increased thickness when compared with their acetone controls (Fig. 1K and 1L).

Epidermal hyperplasia was observed in DMBA/TPA-treated skin in both species at all three time points assessed (Fig. 1I). DMBA/TPA-treated skin was on average 5–6 cell thick compared to the 2-cell thick acetone-treated skin in both species. Quantification of thickness in sections using Image J showed that among DMBA/TPA-treated animals*, Acomys* showed slightly thicker epidermis when compared with C57BL/6 mice (Fig. 1J). An increase in the number of hair follicles was observed in C57BL/6 mice treated with DMBA/TPA at both 12 and 22 weeks, while this was not observed in *Acomys* (Fig. 1I and 1K).

The epidermis consists of the progenitor cells in the basal layer marked by Keratin 14 (KRT14), the spinous layer marked by KRT10, and the cornified envelope marked by Loricrin (LOR). We performed immunohistochemistry for KRT10 (Fig. S3A) and Loricrin (Fig. S3D) and found that the epidermal hyperplasia seen in DMBA/TPA-treated animals in both species may have resulted from the expansion of the layers of the epidermis. The thickness of the KRT10 + spinous layer (Fig. S3A and S3B), and Loricrin + cornified epithelium (Fig. S3D and S3E) significantly increased in DMBA/TPA-treated skin in both species, when compared with their acetone-treated controls, at most time points assessed. This suggests that proliferation and differentiation of the epithelial layers increased upon DMBA/TPA treatment in both species. Fluorescence intensity for KRT10 and Loricrin as a proxy for expression levels did not show significant changes in *Acomys* (Fig. S3C and S3F). Loricrin expression increased at 12 weeks in DMBA/TPA-treated C57BL/6 mice (Fig. S3F) when compared with acetone. Overall, though minor alterations were evident in the two epidermal layers tested on DMBA/TPA treatments, the overall trend was unremarkable between the two species.

No significant differences in number of cells expressing Proliferating cell nuclear antigen (PCNA), a marker for cell proliferation, were observed between DMBA/TPA-treated *Acomys* and C57BL/6 mice skin (Fig. S3G and S3H). We think that while increased epithelial layer thickness upon DMBA/TPA treatment suggests a proliferative response, this likely occurs at timepoints earlier than 12 weeks. DMBA/TPA, but not acetone-treated skin of both species showed γH2AX-positive cells at all three timepoints (Fig. S3J). Variation in the percentage of γH2AX positive cells was observed among the treated animals within both species, and overall no significant differences emerged between DMBA/TPA-treated *Acomys* and mice for γH2AX at both 12 and 22 weeks (Fig. S3I).

Collectively, these results indicate that tumor formation in response to DMBA-TPA treatment is significantly delayed in *Acomys* compared to C57BL/6 mice. Furthermore, the tumors formed in *Acomys* show a distinct benign profile. Tumors in C57BL/6 mice were squamous cell carcinomas while *Acomys* formed benign sebaceous adenomas.

As *Acomys* cells required the same number of oncogenic hits for transformation as mouse cells but *in vivo Acomys* did not form malignant tumors, we speculated that non-cell autonomous mechanisms such as immune surveillance may be responsible for the resistance to tumorigenesis observed in *Acomys*. We assessed immune response in the skin samples using CD3 and CD11b antibodies which identify T cells and macrophages, respectively. CD3^+^ and CD11b^+^ cells accumulated beneath the epidermis upon treatment with DMBA/TPA. *Acomys* showed an enhanced immune response when compared with C57BL/6 mice at most timepoints assessed. Significantly higher infiltration of CD3^+^ T cells (Figs. 1M and S3K) and CD11b^+^ macrophages (Figs. 1N and S3L) occurred in *Acomys* at both 12 and 22 weeks when compared with C57BL/6 mice upon treatment with DMBA/TPA. We also observed that *Acomys,* but not C57BL/6 mice, showed an immune response in acetone-treated skin suggesting that *Acomys* skin may be sensitive to acetone used as treatment control. Despite this, CD3^+^ and CD11b^+^ cells in DMBA/TPA-treated *Acomys* skin were higher than in acetone-treated skin, and significantly higher than in C57BL/6 mice.

Caspase 3-positive cells were higher in DMBA/TPA-treated *Acomys* skin at week 30 when compared with its acetone control. At week 12 and 22, *Acomys* showed similar number of caspase 3^+^ cells in acetone control and DMBA/TPA treatment. In contrast, C57BL/6 mouse skin did not show any caspase 3-positive cells in treatment and control at all time points (Figs. 1O and S3M).

We performed bulk RNA-seq on acetone and DMBA/TPA-treated skin samples from *Acomys* and C57BL/6 mice at week 12 when both species had not yet developed tumors (Data S4). Principal Component Analysis (PCA) showed that the samples separated by species and treatments. DMBA/TPA and acetone- treated skin samples from C57BL/6 mice separated farther from each other than those of *Acomys*, suggesting that the effect of DMBA/TPA on mice was more pronounced (Fig. 2A). This was also evident in the higher number of differentially expressed (DE) genes in mice (Fig. 2B).

**Figure 2.**
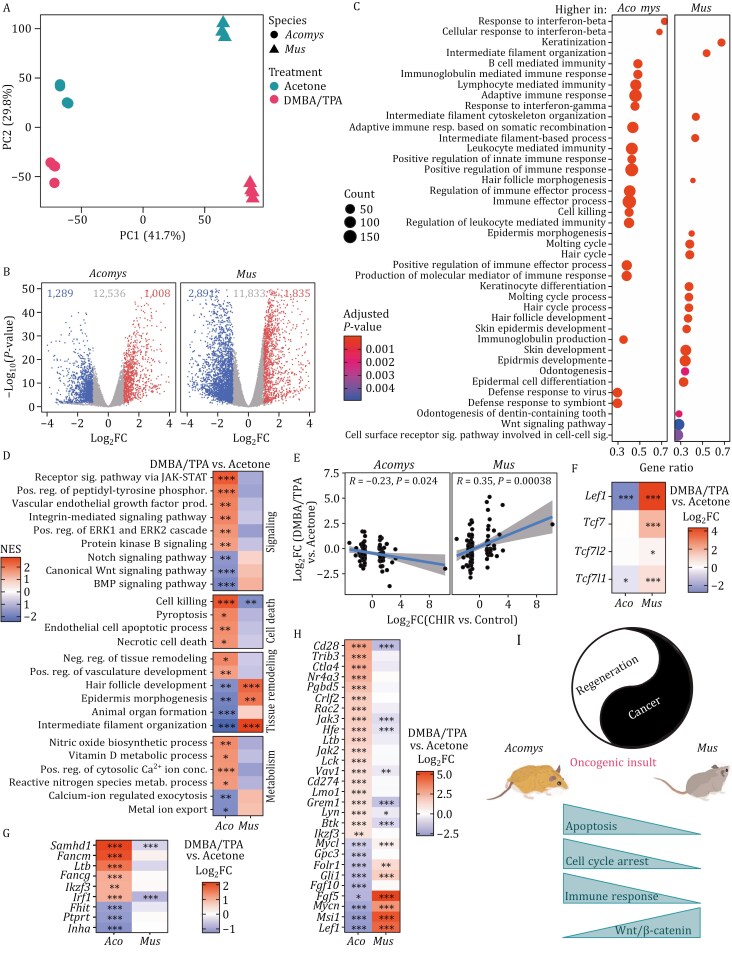
**Transcriptome analysis of DMBA/TPA-treated skin of *Acomys* and mice at week 12**. (A) PCA plot showing variation in gene expression between species and treatments. (B) Volcano plots showing the number of differentially expressed (DE) genes from bulk RNA-seq in mice and *Acomys* skin treated with acetone and DMBA/TPA at week 12. (C) GSEA analysis showing top 20 significantly upregulated and highly enriched terms in skin of *Acomys* and C57BL/6 mice treated with DMBA/TPA at week 12. GSEA was performed on the difference of *Acomys* and mouse log_2_FCs (DMBA/TPA vs. acetone). (D) GSEA analysis showing select GO terms with drastically different response to DMBA/TPA treatment between *Acomys* and C57BL/6 mouse skin at week 12. Here GSEA was performed separately for each species based on log_2_FCs (DMBA/TPA vs. acetone) and the NES and adjusted *P* values are juxtaposed. (E) Comparison of the effects of DMBA/TPA treatment vs. Wnt/β-catenin activation. Wnt target genes and their response to Wnt pathway activator CHIR were obtained from Doumpas et al., 2019. The Pearson correlation is shown. (F) Differential expression of Wnt/β-catenin pathway effector transcription factors in *Acomys* and C57BL/6 mice skin treated with Acetone and DMBA/TPA at week 12. Significance stars are based on adjusted *P* values. (G) Differential expression of known tumor suppressors, from OncoKB database, in DMBA/TPA-treated skin from *Acomys* and C57BL/6 mice at week 12. Only tumor suppressors with drastically different response to DMBA/TPA between *Acomys* and mice are shown. Significance stars are based on adjusted p values. (H) Differential expression of known oncogenes, from OncoKB database, in DMBA/TPA-treated skin from *Acomys* and C57BL/6 mice at week 12. Only oncogenes with drastically different response to DMBA/TPA between *Acomys* and mice are shown. Significance stars are based on adjusted *P* values. (I) Mechanisms contributing to cancer resistance in *Acomys*: Our study indicates that *Acomys* shows resistance to DMBA/TPA-induced squamous carcinogenesis and only shows benign sebaceous adenomas on prolonged treatment. This resistance is associated with increased apoptosis, efficient cell cycle arrest, an enhanced immune response to oncogenic insults, and inhibition of the Wnt/β-catenin signaling in *Acomys* relative to C57BL/6 mice. *P < 0.05, **P < 0.01, ***P < 0.001.

We performed Gene Set Enrichment Analysis (GSEA) on the difference of transcript log_2_ fold changes (log_2_FC) between DMBA/TPA and acetone treated skin samples for both species (Data S5). The enrichment scores calculated by this method represent the degree of upregulation of pathways following DMBA/TPA treatment in *Acomys* when compared with C57BL/6 mice (Fig. 2C). The pathways with stronger upregulation in *Acomys* were predominantly immune related, comprising of multiple immune cell types and signaling pathways (“Response to interferon beta,” “B cell mediated immunity,” “lymphocyte mediated immunity,” “cell killing,” “positive regulation of immune response”). Many interleukins and chemokines were strongly upregulated in DMBA/TPA-treated *Acomys* skin at week 12. Conversely, many of these cytokines were downregulated in mice, supporting the observation of enhanced immune response in *Acomys* (Fig. S4A and S4B). All this corroborated the significantly higher CD3^+^ and CD11b^+^ cells in *Acomys* found by immunohistochemistry (Figs. 1M, 1N, S3K and S3L). DMBA/TPA-treated C57BL/6 mice skin, on the other hand, upregulated genes associated with development and differentiation, like “keratinization,” “keratinocyte differentiation,” “hair follicle development,” “epidermal cell differentiation,” and “hair cycle process” (Fig. 2C).

We further confirmed this result by performing GSEA analysis separately for each species and comparing enrichment scores across species (Data S5), with selected terms shown and grouped by topic (Fig. 2D). As with the previous method, *Acomys* skin showed massive upregulation of immune pathways, while C57BL/6 mouse skin predominantly showed upregulation of terms related to epidermal morphogenesis (Figs. 2D and S4A).

Our GSEA analysis showed that DMBA/TPA-treated skin upregulated JAK-STAT pathway, ERK1 and ERK2 cascade, integrin-mediated signaling pathways, and protein kinase B signaling, whereas Notch, Wnt/β-catenin, and BMP signaling pathways were significantly downregulated in *Acomys* when compared with C57BL/6 mice. Pathways pertaining to “cell killing,” and “apoptosis” were also significantly upregulated in DMBA/TPA-treated *Acomys* skin when compared with C57BL/6 mice, as early as 12 weeks (Fig. 2D). Collectively, these results indicate that *Acomys* shows greatly enhanced immune and apoptotic responses to DMBA/TPA which are likely to confer higher resistance to tumorigenesis.

Wnt/β-Catenin signaling pathway is involved in development, differentiation, regeneration, and cancer. It is required for differentiation of multipotent stem cells in skin and implicated in wound repair and fibrogenesis. Both the induction of tumorigenesis by TPA and the maintenance of cutaneous cancer stem cells have been shown to depend on activation of the Wnt/β-catenin signaling pathway (Su et al., 2018).

Among the terms enriched in DMBA/TPA-treated C57BL/6 mouse skin, “Wnt signaling pathway” was significantly highly upregulated in DMBA/TPA-treated C57BL/6 mouse skin and not in *Acomys* (Fig. 2C). High confidence Wnt/β-catenin target genes (Doumpas et al., 2019) were significantly elevated in DMBA/TPA-treated C57BL/6 mouse skin when compared with *Acomys* skin (Fig. 2E). Among the four effector transcription factors (TFs) of Wnt/β-catenin signaling pathway, *Lef1*, *Tcf7*, and *Tcf7l2* are activators while *Tcf7l1* functions as a repressor (Clevers, 2006). All four TFs were upregulated in mice. Specifically, expression levels of *Lef1* were > 4-fold upregulated in mice and > 2-fold downregulated in *Acomys* (Fig. 2F).

Upon treatment with DMBA/TPA, C57BL/6 mice formed pre-malignant squamous tumors while *Acomys* formed sebaceous adenomas (Fig. 1K and 1L). Sebaceous tumors in humans harbor mutations in LEF1, and in mice are associated with N-terminal mutations in β-catenin, resulting in downregulation of the canonical Wnt/β-catenin pathway (Takeda et al., 2006). This suggests that downregulation of Wnt/β-catenin-*Lef1* may explain the development of benign adenomas rather than malignant carcinomas in *Acomys*.

Lastly, we investigated upstream transcriptional regulators of *Lef1* in both species that may explain the large differences in its transcript levels in response to DMBA/TPA. Wnt/β-catenin effector TF *Lef1* was > 2-fold downregulated in *Acomys* and > 4-fold upregulated in C57BL/6 mice (Fig. 2F). We scanned the *Lef1* promoters (2,000 bp upstream the TSS) from both species for TF bindings sites (Fig. S5; Data S6). While the promoters were fairly conserved, several motifs were exclusively found in either mice or *Acomys* promoter. Among these, we focused on TFs with radically different responses to DMBA/TPA treatment between the two species. Such transcription factors whose motifs only matched in the *Acomys Lef1* promoter include *Tbx21* which was upregulated, and *FoxE1*, *FoxQ1*, and *Dlx2*, which were downregulated in DMBA/TPA-treated *Acomys*. Among these, FoxQ1 and Dlx2 are known to activate Wnt/β-catenin signaling and thus their downregulation may contribute to reduced *Lef1* expression and inhibition of Wnt/β-catenin signaling in *Acomys*. TF whose motifs only matched in the mouse *Lef1* promoter include *Stat1*, *Ikzf3*, *Gli1*, and *n-Myc*. *Gli1* and *n-Myc* were upregulated in DMBA/TPA-treated mice (Fig. S5). Gli1 is a transcriptional effector of the Hedgehog pathway. Since both the oncogenes *n-Myc* and *Gli1*, are targets of Wnt/β-catenin signaling and act to transcriptionally upregulate *Lef1* (Diao et al., 2018; Hao et al., 2019) it is possible that upregulation of these oncogenes may further reinforce and maintain upregulation of Wnt/β-catenin signaling in mice but not in *Acomys.*

We identified additional established oncogenes and tumor suppressors from OncoKB database that are regulated drastically differently between *Acomys* and C57BL/6 mice at 12 weeks in response to DMBA/TPA. The tumor suppressors that were upregulated in *Acomys* and downregulated in C57BL/6 mice included Sterile α motif and HD domain-containing protein 1 (*Samhd1*) which is significantly downregulated in cutaneous T-cell lymphoma; Fanconi anemia genes, *Fancm* and *Fancg*, involved in DNA crosslink repair; lymphotoxin beta (*Ltb*), an immune checkpoint gene for tumor-associated macrophages, Ikaros gene (*Ikzf3*) important for lymphocyte development; and interferon regulatory factor 1 (*Irf-1*), a negative regulator of cell proliferation (Fig. 2G). Additionally, three oncogenes were more than 4-fold upregulated in C57BL/6 mice but > 2-fold downregulated in *Acomys*. These were fibroblast growth factor 5 (*Fgf5)* expressed in melanoma, Mushashi 1 (*Msi1*) an RNA binding protein that regulates apoptosis, differentiation and proliferation; and lymphoid enhancer factor *(Lef1)*, the key transcription factor in Wnt/β-catenin signaling involved in development, regeneration and cancer, and highly downregulated in *Acomys* in this study (Fig. 2H).

Collectively, these results reveal that Wnt/β-catenin pathway, and several oncogenes and tumor suppressor proteins are differentially regulated in *Acomys* compared to C57BL/6 mice in response to oncogenic insult. Wnt/β-catenin pathway and its effectors are strongly downregulated in *Acomys* which is likely to contribute to reduced tumorigenesis. Furthermore, lower levels of *Lef1* transcription factor in *Acomys* may explain the distinct benign tumor profile observed in this species.

Wnt/β-catenin signaling is an important regulator in embryonic development and adult tissue homeostasis. Wnt/β-catenin was proposed to play a role in hair follicle regeneration in *Acomys* (Seifert et al., 2012). However, aberrant activation of Wnt/β-catenin signaling is associated with multiple diseases, that involve cell proliferation and inflammation, such as cancer, cardiovascular disease, lung disease, liver disease, neurodegenerative disease, and therapeutic inhibitors of Wnt/β-catenin pathway, are being actively developed. The basal level of Wnt/β-catenin pathway was higher in *Acomys*, however, upon oncogenic insult *Acomys* inhibited Wnt/β-catenin pathway while C57BL/6 mouse upregulated it (Fig. S5B). We hypothesize that *Acomys* has evolved a tighter control of Wnt/β-catenin pathway to prevent tumorigenesis during regeneration. One mechanism for this may be via the tumor suppressor, p53 which in our study had relatively higher influence than pRb in preventing malignant transformation in *Acomys* fibroblasts.

As *Acomys* lifespan is twice longer than that of laboratory mice, relatively older age of mice during carcinogenic treatment may contribute to higher cancer susceptibility. However, while we were writing this manuscript, two pre-prints reported that even when very young (2–3 months old) animals were used *Acomys* was more resistant to DMBA/TPA-mediated carcinogenesis (Andrew White et al., 2024; Vitorino et al., 2024). While these studies are generally consistent with our results, the papilloma development in *Acomys* was either not observed or occurred much later than in our study. One possible explanation is that these studies used very young *Acomys* while we used animals older than 1 year of age.

While *Acomys* cells required the same number of oncogenic hits for malignant transformation, *in vivo Acomys* showed higher cancer resistance relative to laboratory mice. A likely explanation is that additional protection in *Acomys* was conferred *in vivo* by cell non-autonomous mechanisms. In conclusion, we report that *Acomys* shows higher resistance to cancer mediated by a combination of cell-autonomous mechanisms such as more robust cell cycle arrest and apoptosis, and non-cell-autonomous mechanisms, such as heightened immune response and downregulation of Wnt/β-catenin signaling (Fig. 2I). As strategies to improve regenerative potential in human patients are being actively researched, the knowledge of naturally evolved compensatory anticancer mechanisms in a regenerative mammal may provide therapeutic targets for modulation to prevent tumor formation during regenerative therapies. For example, selective Wnt/β-catenin pathway inhibitors might be explored as a part of regenerative therapies.

## Supplementary data

Supplementary data is available at *Protein & Cell* online https://doi.org/10.1093/procel/pwaf024.

pwaf024_suppl_Supplementary_Materials

## Footnotes

The authors would like to thank Cheyenne Rechsteiner for her help assembling the figures for publication.

A.S. and V.G. directed the study; F.A., A.Sh., V.B., A.S., and V.G. designed experiments; F.A., I.F., I.S, S.J.K., and M.L performed experiments.; F.M. performed Bioinformatic analysis; F.A., F.M., X.L., A.Sh., V.B., A.S., and V.G. interpreted the data: F.A., F.M., A.S., and V.G. wrote the manuscript with input from all authors. F.A. and F.M. contributed equally to the study. No AI-assisted technologies were used in any part of this work.

V.G. is a member of Scientific Advisory Boards of GenFlow Bio, DoNotAge.org, Elysium, Matrix Bio, Faunsome, BellSant, and WndrHlth. The other authors declare no conflict of interest.

This work is supported by grants from the US National Institute on Aging to V.G. and A.S.

All institutional guidelines for the care and use of laboratory animals were followed. All animal experiments were approved and performed in accordance with guidelines set forth by the University of Rochester Committee on Animal Resources with protocol number 2017-033 (mouse) and 2017-027 (*Acomys dimidiatus*, African spiny mice).

All authors have read and approved the manuscript for publication.

The data, analytic methods, and study materials will be made available to other researchers for purposes of reproducing the results or replicating the procedure. The RNA-seq raw data and count tables have been deposited in GEO (GSE287010). Code and additional files are available on GitHub.
