# Supplementary material for: African spiny mice show resistance to DMBA/TPA-induced squamous carcinogenesis with distinct benign tumor profile
Source: Protein Cell. 2025 Mar 22;16(10):896–904. doi: 10.1093/procel/pwaf024 (PMC12578293; doi:10.1093/procel/pwaf024)
Supplement: pwaf024_suppl_Supplementary_Materials [file pwaf024_suppl_supplementary_materials.zip › pwaf024_suppl_Supplementary_Figures_S1-S5_Datas_S1.pdf]

SUPPLEMENTARY MATERIALS

African spiny mice show resistance to DMBA/TPA-induced squamous carcinogenesis with distinct benign tumor profile

Fathima Athar[1, ⌘], Francesco Morandini[1, ⌘], Iqra Fatima[2], Isabella Silvestri[1], Sei Joong Kim[1], Minseon Lee[1], Xiaoyan Liao[3], Andrei Sharov[2], Vladimir Botchkarev[2], Andrei Seluanov[1#], Vera Gorbunova[1#]

[1]Department of Biology, University of Rochester, Rochester, NY 14627, USA

[2]Department of Dermatology, Boston University, Boston, MA 02118, USA

[3]Department of Pathology and Laboratory Medicine, University of Rochester Medical Center, Rochester, NY 14642, USA

⌘Equal contribution

#Correspondence:

Vera.Gorbunova@rochester.edu

Andrei.Seluanov@rochester.edu

Table of contents:

Figure S1

Figure S2

Figure S3

Figure S4

Figure S5

Methods

References

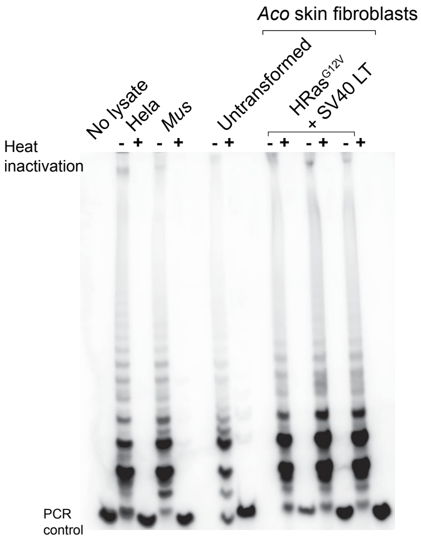

Figure S1: *Acomys* skin fibroblasts are positive for telomerase activity

Telomere Repeat Amplification Protocol (TRAP) assay using untransformed skin fibroblasts from *Acomys* (*Aco*), laboratory mice (*Mus*) and transformed fibroblasts from *Acomys* expressing HRasG12V+SV40 LT. Hela cells were used as positive control. Each reaction contained an internal PCR control. Heat inactivation (+/-) inactivates telomerase activity.

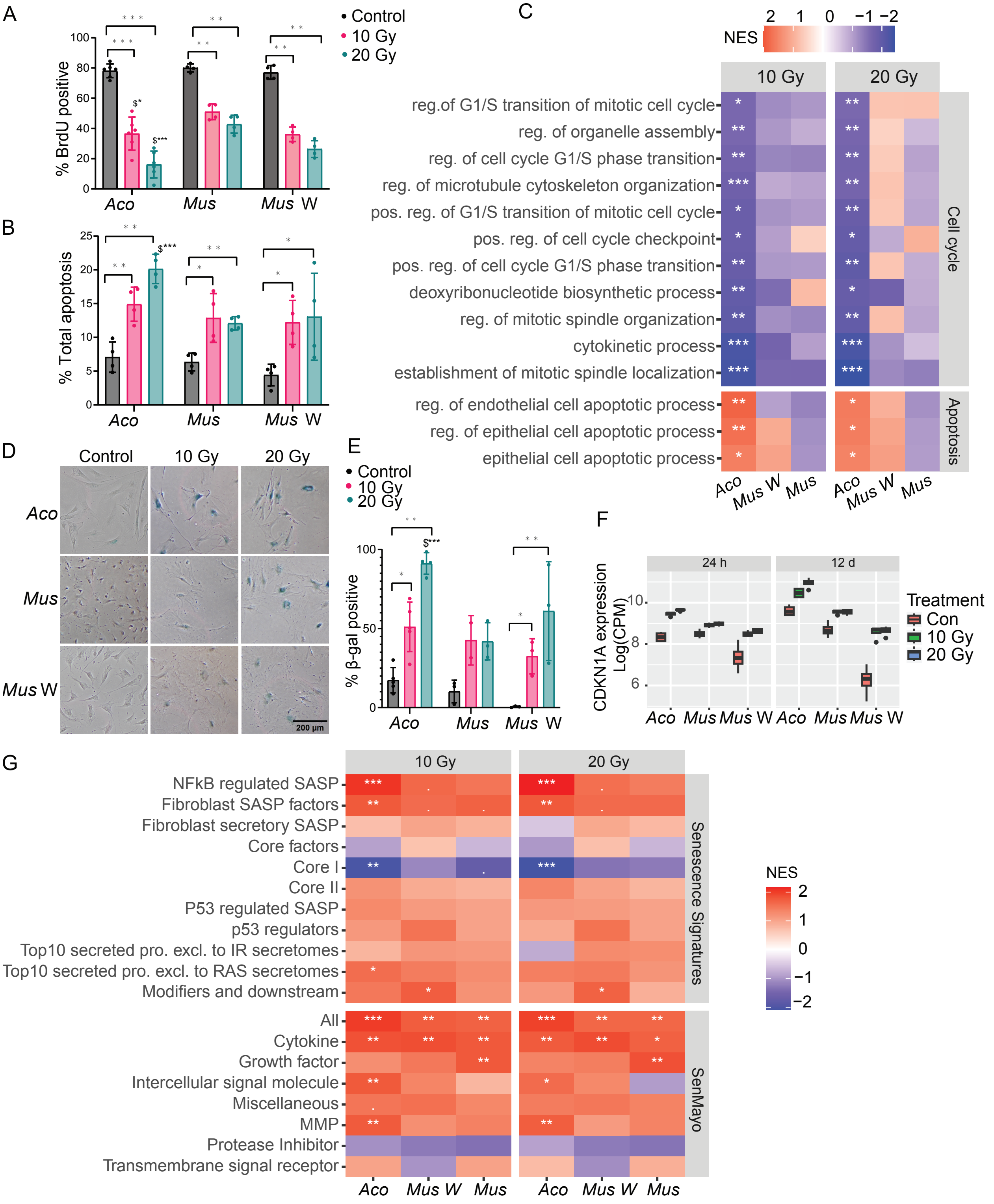

Figure S2: *Acomys* shows robust cell cycle arrest and senescence response compared to laboratory mice

(legend continued on next page)

(A) BrdU assay in skin fibroblasts of *Acomys* (*Aco*), laboratory mice (*Mus*), and wild-caught mice (*Mus W*) 3 days after treatment with γ-radiation. Data is represented as mean ± SD. Significance from two-tailed paired t-tests are indicated. Significance between *Acomys* and *Mus*, for similar conditions, using two-tailed unpaired t-test is shown with '$'.

(B) Apoptosis assay in skin fibroblasts of *Acomys* (*Aco*), laboratory mice (*Mus*) and wild-caught mice (*Mus W*), 3 days after treatment with γ-radiation. Data is represented as mean ± SD. Significance from two-tailed paired t-tests is indicated. Significance between *Acomys* and *Mus*, for similar conditions, using two-tailed unpaired t-test is shown with '$'.

(C) GSEA analysis of skin fibroblasts from *Acomys* (*Aco*), wild-caught mice (*Mus W*), and laboratory mouse (*Mus*) 24 h post-γ-radiation. Select terms corresponding to cell cycle and apoptosis, differentially enriched between *Acomys* and mouse are shown. Significance stars are based on adjusted p values.

(D) Representative images from Senescence associated β-galactosidase (SA β-gal) assay showing stained senescent cells in skin fibroblasts of *Acomys*, laboratory mice (*Mus*), and wild-caught mice (*Mus W*), 12 days after treatment with γ-radiation. Scale, 200 μm.

(E) Quantification of SA-β-galactosidase assay. Data is represented as mean ± SD. Significance from two-tailed paired t-tests is indicated. Significance between *Acomys* and *Mus*, for similar conditions, using two-tailed unpaired t-test is shown with '$'.

(F) CDKN1A (p21) normalized transcript levels in γ-radiation-treated fibroblasts from *Acomys* (*Aco*), laboratory mice (*Mus*), and wild-caught mice (*Mus W*) at 24 h and 12 days post-radiation.

(G) GSEA of Senescence Associated Secretory Phenotype (SASP) in skin fibroblasts from *Acomys*, wild-caught mice (*Mus W*), and laboratory mice (*Mus*) 12 days post-γ-radiation. Significance stars are based on adjusted p values. * $p < 0.05$, ** $p < 0.01$, *** $p < 0.001$.

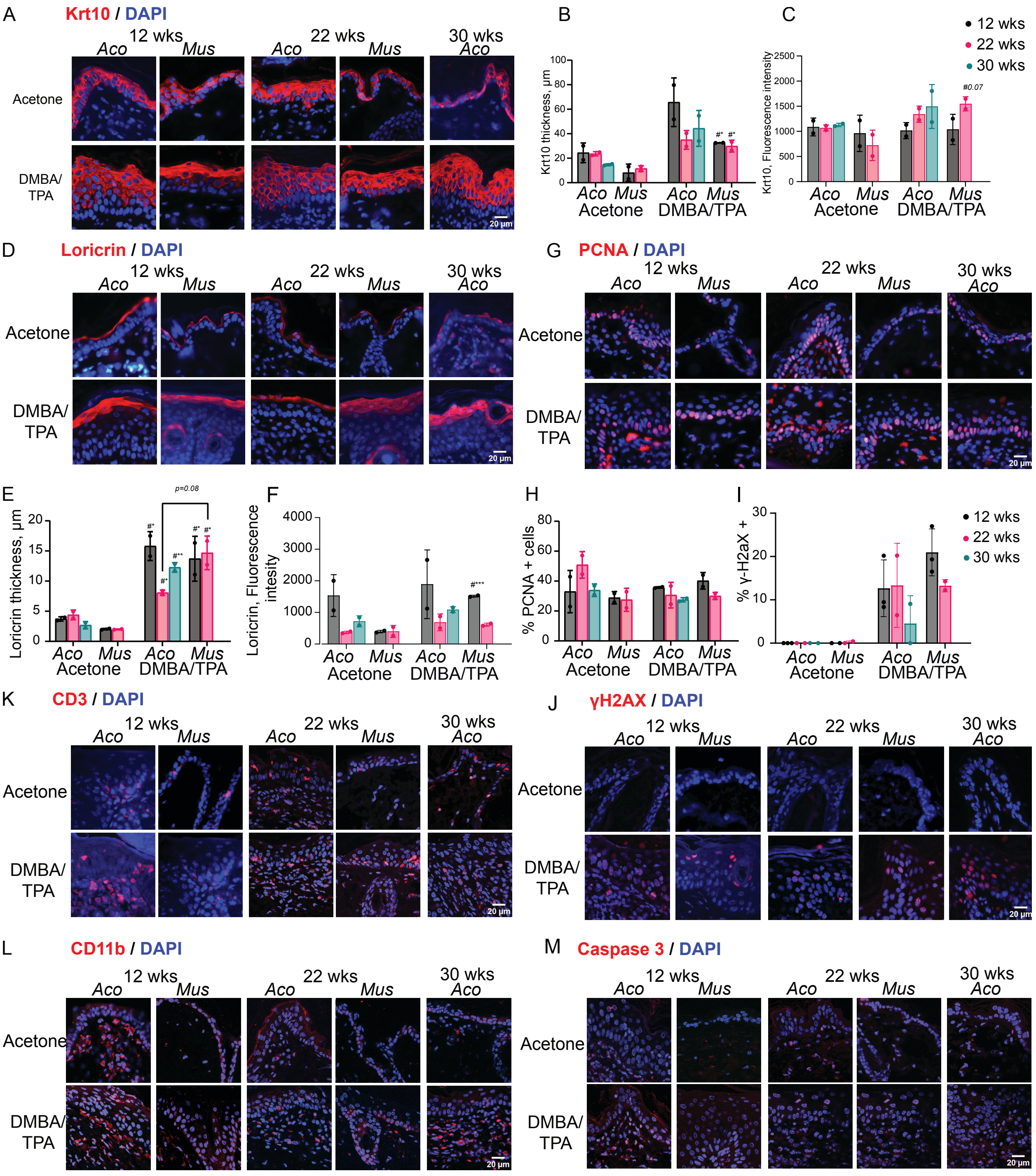

Figure S3: Immunohistochemistry of skin from *Acomys* and C57BL/6 mice treated with acetone and DMBA/TPA at 12, 22, and 30 weeks

(A) Representative images of Krt10 in skin sections from animals treated with DMBA/TPA and acetone at 12, 22 and 30 weeks. Scale, 20 µm.

(legend continued on next page)

(B) Analysis and quantification of Krt10 layer thickness using Image J. No significant changes were observed.

(C) Quantification of fluorescence intensity Krt10 staining using Image J. Significance between *Acomys* and *Mus*, for similar conditions, using two-tailed unpaired t-test is shown with ‘#’.

(D) Representative images of Loricrin in skin sections from animals treated with DMBA/TPA and acetone at 12, 22 and 30 weeks. Scale, 20 µm.

(E) Analysis and quantification of Loricrin layer thickness using Image J

(F) Quantification of fluorescence intensity Loricrin staining using Image J. Significance between *Acomys* and *Mus*, for similar conditions, using two-tailed unpaired t-test is shown with ‘#’.

(G) Representative images of PCNA in skin sections from animals treated with DMBA/TPA and acetone at 12, 22 and 30 weeks. Scale, 20 µm.

(H) Percentage positive cells of PCNA in skin from animals treated with DMBA/TPA and acetone. No significant changes were observed.

(I) Percentage positive cells of γH2AX in skin from animals treated with DMBA/TPA and acetone. No significant changes were observed.

(J) Representative images of γH2AX in skin sections from animals treated with DMBA/TPA and acetone at 12, 22 and 30 weeks. Scale, 20 µm.

(K) Representative images of immune cells markers, CD3 in skin sections from animals treated with DMBA/TPA and acetone. Scale, 20 µm.

(L) Representative images of immune cells markers, CD11b in skin sections from animals treated with DMBA/TPA and acetone. Scale, 20 µm.

(M) Representative images of Caspase 3 in skin sections from animals treated with DMBA/TPA and acetone. Scale, 20 µm

* $p < 0.05$, ** $p<0.01$, *** $p<0.001$.

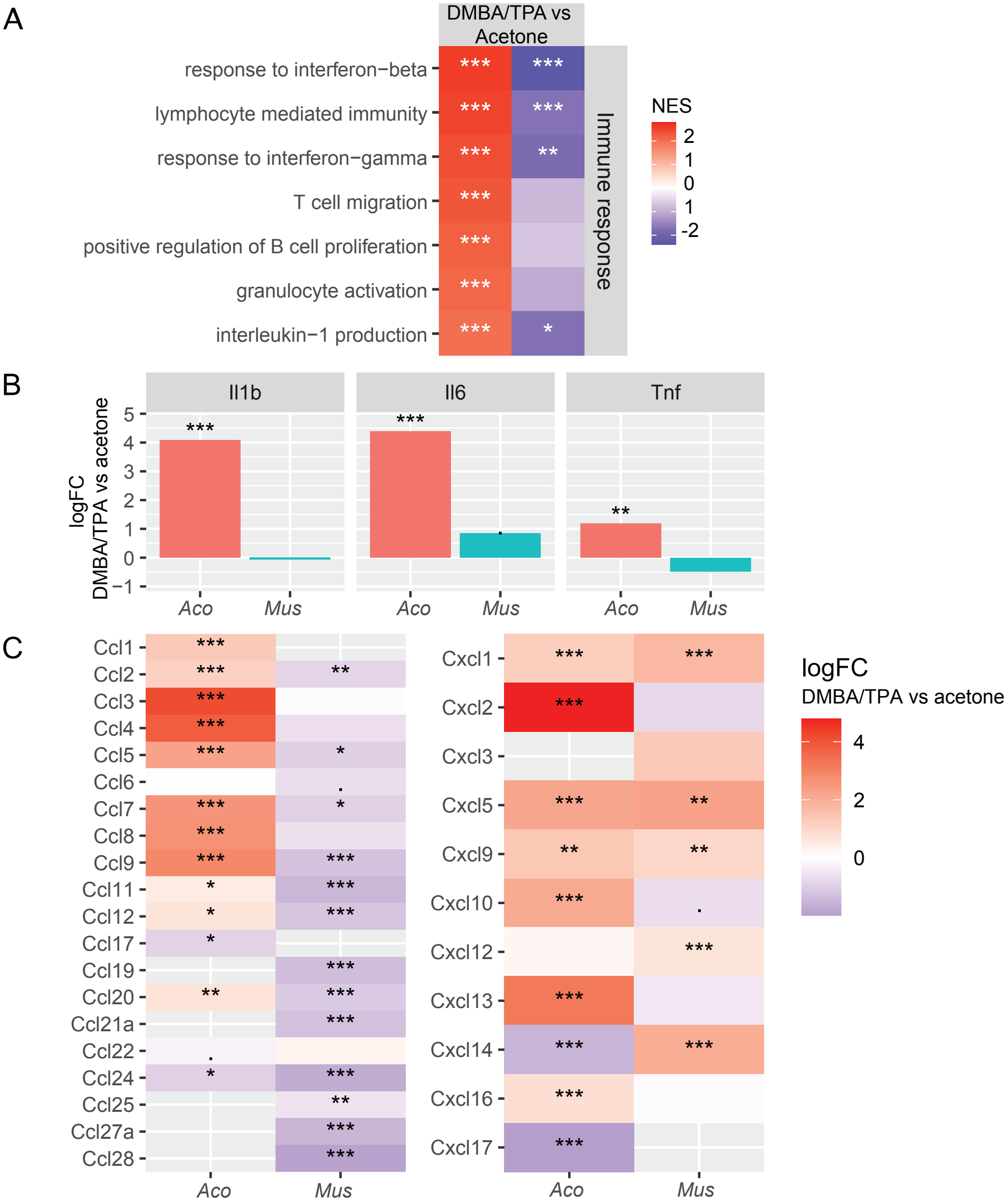

Figure S4: An enhanced immune response in *Acomys* skin in response to DMBA/TPA treatment is associated with increased expression of cytokines and chemokines at week 12 of treatment

(A) GSEA analysis showing GO term, ‘Immune response’ with has drastically different response to DMBA/TPA treatment between *Acomys* and C57BL/6 mouse skin at week 12. GSEA was performed separately for each species based on logFCs (DMBA/TPA vs acetone) and the NES and adjusted p values are juxtaposed.

(B) Expression levels of Il1b, Il6, and TNF-α in skin of *Acomys* and mice treated with acetone or DMBA/TPA at week 12 of treatment. Significance stars are based on adjusted p values.

(C) Expression levels of chemokines in skin of *Acomys* and mice treated with acetone or DMBA/TPA at week 12 of treatment. Absent heatmap cells indicate the gene was not expressed to a detectable level. Significance stars are based on adjusted p values.

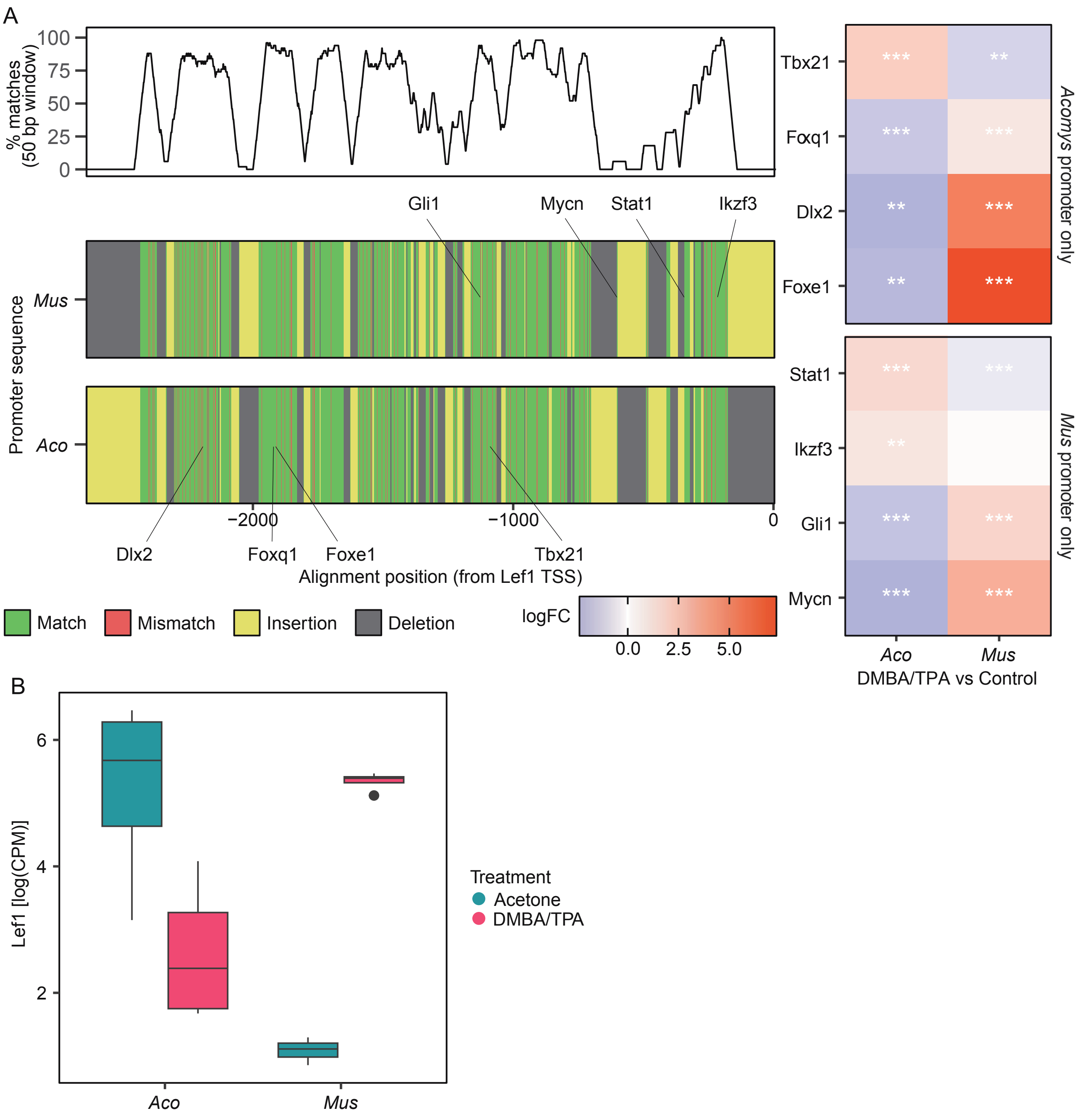

Figure S5: Analysis of Lef1 promoter in *Acomys* and mice

(A) Lef1 promoter conservation between *Acomys* and mice; Differential expression results for TFs whose motifs were exclusively found in either the *Acomys* or mouse Lef1 promoter and with drastically different expression responses to DMBA/TPA treatment between the species. Significance stars are based on adjusted p values.

(B) Lef1 normalized transcript levels in acetone and DMBA/TPA treated *Acomys* and C57BL/6 mice.

METHODS

Animals

All animal experiments were approved and performed in accordance with guidelines set forth by the University of Rochester Committee on Animal Resources with protocol number 2017-033 (mouse) and 2017-027 (*Acomys dimidiatus*, African spiny mice). Both *Acomys dimidiatus* (Riddell, McDonough et al. 2025), hereafter referred to as *Acomys* in the manuscript, and C57BL/6 mice were group housed in IVC cages (up to 5 animals/cage) and fed standard chow diet and water ad libitum. *Acomys* diet was additionally supplemented with sunflower seeds. Animal rooms were maintained at 21–24 °C and 35–75% relative humidity, with 12/12 h (6 a.m. to 6 p.m.) dark–light cycle.

Cell culture

Primary fibroblasts were isolated from dorsal skin of *Acomys* and mice using the protocol described previously in detail (Seluanov, Vaidya et al. 2010). Independent cell lines were derived from three to four individuals from each species. Isolated cells were grown in EMEM (ATCC) containing 15% fetal bovine serum (GIBCO), 100 U/mL penicillin, and 100 mg/mL streptomycin antibiotics (GIBCO). Culture conditions were 37°C with 5% CO_2 and 5% O_2. Cells were passaged at 80-90% confluence. Cells with population doublings lower than 30 were used for all experiments.

Telomerase Repeated Amplification Protocol (TRAP)

TRAP assay was performed using TRAPeze kit (Cat. #S7700, Millipore Sigma). Briefly, 0.5 million cells were resuspended in CHAPs lysis buffer, and the amount of protein was estimated using Pierce BCA Protein Assay Kits (Cat. # 23225, Thermo Scientific). TRAP assay was performed using 250 ng of cell extracts, and manufacturer's instructions were followed. HeLa cell lysates were used as positive controls.

Soft Agar Assay

Following plasmids were used to generate stable cell lines: pCMV-HRas^{G12V} (Clontech), Piggybac vectors- pPB-SV40 LT, pPB-SV40 LT-K1, and pPB-SV40 LT- Δ434-444, pBase, and GFP control plasmid (Launchpad AVA2590, plasmid #85442). Cells were transfected using Amaxa nucleofector (Lonza) following the manufacturer's protocol T-20. Stable cell lines were selected using puromycin (0.25-0.5 µg/mL) or hygromycin (25-50 µg/mL), depending on the plasmids and

their combinations. For soft agar assay, 2X complete medium was prepared using 2X EMEM, 30% FBS, and 2X antibiotics as required. A base layer was poured into plates after mixing 2X complete media with 1% agarose (Difco Agar Noble, Sigma). Then 2X complete medium was mixed with 0.8% agarose and 10,000 cells (for 35 mm well) and layered on top of the base layer. Following solidification of agarose, 1-2 mL of 1X complete media with antibiotics was added on top and refurbished twice a week. Plates were incubated at 37°C in a humidified incubator for three weeks. Colonies in soft agar plates were imaged. To stain the colonies Nitroblue tetrazolium (NBT) chloride solution (1 mg/mL in PBS) was prepared. NBT solution (200 µl/35 mm well) was added to the medium and incubated overnight at 37 °C in CO_2 incubator. Colonies were photographed once stained.

Xenograft assay

Animal experiments were performed under pre-approved protocols and in accordance with guidelines set by the University of Rochester Committee on Animal Resources (UCAR). Fibroblasts stably expressing oncogenes or a combination of oncogenes were tested for tumor formation in nude mice (Charles River, Crl:NIH-Lystbg-JFoxn1nuBtkxid). Using a 22 guage needle, each flank of the nude mice was injected with 2 X 10^6 cells in 100 µL PBS mixed with an equal volume of Matrigel. A total of 14-16 injections per cell line were tested. Tumor formation was monitored twice a week, and its dimensions were measured using Vernier calipers. A tumor long diameter < 5 mm was considered negative, and 20 mm was considered the tumor burden endpoint. Mice that did not reach tumor burden endpoints were terminated after a maximum of 60 days. Euthanized mice were photographed, and tumors were excised, photographed, weighed, parts frozen at -80°C, and preserved in formalin.

Gamma-Radiation

Radiation treatment was performed using γ-radiator (Model 8114 Shepherd Cs^{137}). Cells were seeded 24-48 h before treatments. Following treatment with radiation doses of 10 and 20 Gy, cell culture medium was changed as soon as possible, and cells were returned to the incubator. For induction of senescence, radiation-treated cells were incubated for 12 days, and the culture medium was replaced twice a week. BrdU and apoptosis analysis was done three days post radiation, while β-gal assay was done 12 days post-radiation.

BrdU incorporation assay

Cells were seeded 24 h before radiation treatment. Following radiation cells were allowed to grow in the presence of 3 μg/mL BrdU (BD Pharmingen) for 48 h. Cells were trypsinized and fixed in 70% cold ethanol for 30 min. For staining, fixed cells were washed twice with PBS and treated with 2 N HCl for 30 min for DNA denaturation. Following two more PBS washes, cells were incubated with 5% BSA for 1 h at room temperature (RT). Cells were then incubated overnight at 4°C with anti-BrdU (Alexa Fluor® 647 Mouse anti-BrdU, Clone 3D4 (RUO)) antibody. Following 2X PBS washes, stained cells were analyzed by flow cytometer (Cytoflex S (B49006) Beckman Coulter Inc.) with appropriate positive and negative controls. CytExpert 2.4.0.28 was used to acquire data and analysis was performed using Kaluza Analysis Version 2.1.

Apoptosis assay

Apoptosis assay was performed using Annexin V FLUOS staining kit ((Roche, Cat. No. 11 858 777 001) following manufacturer's instructions. Briefly, culture supernatants, PBS washes, and trypsinized cells were pooled and collected. Following two washes with PBS, the cell pellet was resuspended in Annexin-V-FLUOS labeling reagent containing Annexin V and propidium iodide. Following a 10 min incubation on ice, cells were immediately analyzed using a flow cytometer with appropriate positive and negative controls.

Senescence-associated β-gal staining

γ-radiation-treated cells were incubated for 12 days, and the media was refurbished twice a week. After 12 days, approximately 24-48 h before staining, cells were seeded to be approximately 40% confluent. Cells were washed 2X with PBS and fixed for 5 min with 2% formaldehyde. Cells were washed gently to remove formaldehyde and staining solution (1 mg/ml X-gal in DMSO, 40 mM Citric acid/Sodium phosphate buffer pH 6, 5 mM potassium ferricyanide, 5 mM potassium ferricyanide, 150 mM NaCl, 2 mM $MgCl_2$) was added. Plates were incubated for 12-24 h in a 37°C incubator without CO_2. Following the development of a blue color, cells were washed 2X with PBS, overlayed with 70% glycerol, imaged, and the percentage of SA-β-gal positive cells was determined.

7,12-Dimethylbenz(a)anthracene/12-O-tetradecanoylphorbol-13-acetate (DMBA/TPA) treatment

C57BL/6 mice and *Acomys* aged between 1 -1.5 years were used in this experiment. A minimum of 4-5 animals were used per group for experimental analysis. A single dose of DMBA (7.8 mM dissolved in acetone) was topically applied on the shaved skin of dorsal trunk of the animal under

isoflurane anesthesia. One week after DMBA application, animals were treated with TPA on the same dorsal skin (0.4 mM) twice a week. Animals were sacked at two different time points- 12 weeks and 22 weeks of TPA treatment. 5 (2 Acetone, 3 DMBA/TPA) *Acomys* were continued to be treated for 30 weeks. Animals were re-shaved, and nails trimmed periodically as needed under anesthesia during the course of treatment. Formation of macroscopically visible skin tumors was continuously monitored and their occurrence in maximum number of animals of the group was taken as end point of experiment.

Immunohistochemistry

Paraffin blocks were frozen at -20 °C and 5 µm sections were cut using a microtome with water bath temperature of 42°C for mouse skin and 40°C for spiny mice skin (skin layers were separating/tearing at 42). Slides were baked at 70°C for 30 minutes. (some of the wax melts away). Dewaxing and rehydration were done using the following solution series for 3 minutes each: Xylene 2X, 50 % Xylene-Ethanol, 100 % Ethanol 2X, 90 % ethanol, 70 % ethanol, 50% Ethanol, distilled water 2X and PBS. Antigen retrieval was done at settings---using (Dako Cytomation PTLink). Slides were immediately transferred to PBS without delay to avoid drying. Slides were washed 3X, 5min each in PBS. Sections were removed from PBS and circled with PAP pen. Blocking was performed for 45-60 minutes at room temperature with blocking buffer specific for each antibody. Avidin-Biotin blocking was additionally performed for PCNA followed by blocking with 7% mouse serum. Blocking serum were purchased from Jackson laboratories (Mouse-015-000-120, Goat-005-000-121, Donkey-017-000-121). Overnight incubations were performed with primary antibodies (See Table). Slides were washed 3X for5min each in PBS. Secondary antibody incubations were performed at 37 °C for 45 minutes. Slides were washed 3X for 5 min each. Briefly for 1 s slides were dipped in Triton (0.05%) solution for efficient draining of PBS and mounted in DAPI Vectashield Antifade mounting medium (mounting medium-H-1900-10ml) add DAPI (10 µM) 3ul. Primary antibodies diluted in 1% BSA, secondary antibodies were diluted in PBS. For all staining except Caspase3, antigen retrieval was done at low pH. Antibodies used are as follows: PCNA (Biolegend #307904, clone PC10), p-H2AX (Cell Signaling, Clone S139, #9718S), Loricrin (Biolegend, #905104, Poly19051), Krt10 (Biolegend, #905401, Clone PRB-159p), CD3 (, Biorad, #MCA1477), CD11b (Abclonal, #A1581), Caspase 3 (Abcam #AB3847), Alexa555 (Invitrogen, #A21434), TRITC (Invitrogen, #A31572).

Bulk RNA-seq from fibroblasts and whole skin tissues of acetone and DMBA/TPA treated animals

Total RNA was extracted from fibroblasts or skin using PureLinkTM RNA Mini Kit (Thermo Fisher Scientific) following manufacturer's instructions. mRNA Stranded libraries (single-end, 100 bp, for fibroblasts and Total RNA Stranded libraries (paired-end, 150 bp, for skin) were sequenced using NovaSeq 6000 at the Genomics Research Center (GRC), University of Rochester, NY.

Reads were trimmed using TrimGalore! and aligned to the *M. musculus* and *Acomys* genomes using Hisat2 (Kim, Paggi et al. 2019), removing multimapping reads with Samtools (Liao, Smyth et al. 2014). The M. musculus genome and gene annotations were sourced from ENSEMBL (GRCm39, ENSEMBL build 108). For *Acomys* we used the genome and gene annotations produced by Nguyen et al (Nguyen, Fard et al. 2023). Alignments were then counted using featureCounts (Liao, Smyth et al. 2014). 1 wild-caught mouse sample from the irradiation experiment and 1 laboratory mouse sample from the DMBA/TPA experiment were exclude due low mapping rate compared to other samples in the same experiment. Throughout our analysis we matched *Acomys* genes to *M. musculus* gene by the gene symbols provided by Nguyen et al., as their original gene annotation was derived from *M. musculus* annotations. Differential expression testing was performed with EdgeR (Robinson, McCarthy et al. 2010) and GSEA enrichment was performed with ClusterProfiler (Wu, Hu et al. 2021), using gene sets obtained from Gene Ontology, MSigDB (SenMayo), OncoKB, TSGene or manually curated. When comparing the response of *Acomys* and M. musculus to irradiation or DMBA/TPA treatment, we first performed differential expression testing and GSEA within the species and then comparing statistics (expression log fold-changes (logFC), normalized enrichment scores (NES) and p values) across species. Specifically, counts for each species were filtered to remove low expression genes using filterByExpr, normalized using the TMM method and compared across treatments (10 gy/ 20 gy vs untreated, DMBTA/TPA vs acetone treated). In the irradiation experiment we tested the 24 h and 12 d time points separately as they were sequenced in separate batches and additionally included a cell line covariate. When comparing baseline levels of gene expression across species, we performed the same steps on a count matrix containing only control samples. To identify genes and gene sets with drastically different responses to treatment across *Acomys* and M. musculus we systematically filtered to those significantly upregulated/downregulated in *Acomys* and either not significantly upregulated/downregulated in M. musculus or significantly regulated in the opposite direction as *Acomys*. In the irradiation experiment, these conditions were imposed for both radiation doses. We additionally investigated differential regulation between *Acomys* and *M. musculus* in response to DMBA/TPA by performing GSEA on a score calculated as logFC_Acah_DTvsC – logFC_Mmus_DTvsC.

Motif analysis

2000 bp upstream of the *Acomys* and *M. musculus* Lef1 TSS were scanned for transcription factor (TF) binding sites using FIMO(Grant, Bailey et al. 2011), with the JASPAR 2022 core vertebrate non-redundant motif database(Castro-Mondragon, Riudavets-Puig et al. 2022). We used the p-value cutoff of 1e-4 and only considered the best match for a given TF when more than one match was present. Next, we restricted to TF whose binding motifs had a matching sequence in either the *Acomys* or M. musculus Lef1 promoter but not both. Among these, we considered TFs to be potentially involved in the differences in Lef1 regulation seen when comparing *Acomys* and *M. musculus* if expression of the TF itself responded drastically differently to DMBA/TPA treatment between *Acomys* and *M. musculus*.

References

Castro-Mondragon, J. A., R. Riudavets-Puig, I. Rauluseviciute, R. B. Lemma, L. Turchi, R. Blanc-Mathieu, J. Lucas, P. Boddie, A. Khan, N. Manosalva Perez, O. Fornes, T. Y. Leung, A. Aguirre, F. Hammal, D. Schmelter, D. Baranasic, B. Ballester, A. Sandelin, B. Lenhard, K. Vandepoele, W. W. Wasserman, F. Parcy and A. Mathelier (2022). "JASPAR 2022: the 9th release of the open-access database of transcription factor binding profiles." Nucleic Acids Res **50**(D1): D165-D173.

Grant, C. E., T. L. Bailey and W. S. Noble (2011). "FIMO: scanning for occurrences of a given motif." Bioinformatics **27**(7): 1017-1018.

Kim, D., J. M. Paggi, C. Park, C. Bennett and S. L. Salzberg (2019). "Graph-based genome alignment and genotyping with HISAT2 and HISAT-genotype." Nat Biotechnol **37**(8): 907-915.

Liao, Y., G. K. Smyth and W. Shi (2014). "featureCounts: an efficient general purpose program for assigning sequence reads to genomic features." Bioinformatics **30**(7): 923-930.

Nguyen, E. D., V. N. Fard, B. Y. Kim, S. Collins, M. Galey, B. R. Nelson, P. Wakenight, S. M. Gable, A. McKenna, T. K. Bammler, J. MacDonald, D. M. Okamura, J. Shendure, D. R. Beier, J. M. Ramirez, M. W. Majesky, K. J. Millen, M. Tollis and D. E. Miller (2023). "Genome Report: chromosome-scale genome assembly of the African spiny mouse (Acomys cahirinus)." G3 (Bethesda) **13**(10).

Riddell, B., M. McDonough, A. Ferguson, J. M. Kimani, T. R. Gawriluk, C. Peng, S. G. Kiama, V. O. Ezenwa and A. W. Seifert (2025). "Complex tissue regeneration in Lophuromys reveals a phylogenetic signal for enhanced regenerative ability in deomyine rodents." Proc Natl Acad Sci U S A **122**(1): e2420726122.

Robinson, M. D., D. J. McCarthy and G. K. Smyth (2010). "edgeR: a Bioconductor package for differential expression analysis of digital gene expression data." Bioinformatics **26**(1): 139-140.

Seluanov, A., A. Vaidya and V. Gorbunova (2010). "Establishing primary adult fibroblast cultures from rodents." J Vis Exp(44).

Wu, T., E. Hu, S. Xu, M. Chen, P. Guo, Z. Dai, T. Feng, L. Zhou, W. Tang, L. Zhan, X. Fu, S. Liu, X. Bo and G. Yu (2021). "clusterProfiler 4.0: A universal enrichment tool for interpreting omics data." Innovation (Camb) **2**(3): 100141.
